# Immunophenotype‐associated gene signature in ductal breast tumors varies by receptor subtype, but the expression of individual signature genes remains consistent

**DOI:** 10.1002/cam4.4095

**Published:** 2021-06-29

**Authors:** Michael Behring, Yuanfan Ye, Amr Elkholy, Prachi Bajpai, Sumit Agarwal, Hyung‐Gyoon Kim, Akinyemi I. Ojesina, Howard W Wiener, Upender Manne, Sadeep Shrestha, Ana I. Vazquez

**Affiliations:** ^1^ Department of Epidemiology University of Alabama at Birmingham Birmingham AL USA; ^2^ Department of Pathology and Surgery University of Alabama at Birmingham Birmingham AL USA; ^3^ Comprehensive Cancer Center University of Alabama at Birmingham Birmingham AL USA; ^4^ Department of Epidemiology and Biostatistics Michigan State University East Lansing MI USA; ^5^ Institute for Quantitative Health Science & Engineering East Lansing MI USA

**Keywords:** breast cancer, microenvironment, transcriptomics, tumor‐infiltrating immune cells

## Abstract

**Background:**

In silico deconvolution of invasive immune cell infiltration in bulk breast tumors helps characterize immunophenotype, expands treatment options, and influences survival endpoints. In this study, we identify the differential expression (DE) of the LM22 signature to classify immune‐rich and ‐poor breast tumors and evaluate immune infiltration by receptor subtype and lymph node metastasis.

**Methods:**

Using publicly available data, we applied the CIBERSORT algorithm to estimate immune cells infiltrating the tumor into immune‐rich and immune‐poor groups. We then tested the association of receptor subtype and nodal status with immune‐rich/poor phenotype. We used DE to test individual signature genes and over‐representation analysis for related pathways.

**Results:**

*CCL19* and *CXCL9* expression differed between rich/poor signature groups regardless of subtype. Overexpression of *CHI3L2* and *FES* was observed in triple negative breast cancers (TNBCs) relative to other subtypes in immune‐rich tumors. Non‐signature genes, *LYZ*, *C1QB*, *CORO1A*, *EVI2B*, *GBP1*, *PSMB9*, and *CD52* were consistently overexpressed in immune‐rich tumors, and *SCUBE2* and *GRIA2* were associated with immune‐poor tumors. Immune‐rich tumors had significant upregulation of genes/pathways while none were identified in immune‐poor tumors.

**Conclusions:**

Overall, the proportion of immune‐rich/poor tumors differed by subtype; however, a subset of 10 LM22 genes that marked immune‐rich status remained the same across subtype. Non‐LM22 genes differentially expressed between the phenotypes suggest that the biologic processes responsible for immune‐poor phenotype are not yet well characterized.

## Graphical abstract

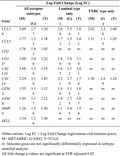

## 1 INTRODUCTION

The immune system has a strong influence on both tumorigenesis and clinical outcomes in all cancers.^1^ Recent discoveries have led to the development of new immune‐based prognostic markers and innovative therapy. However, host antitumor immune response is a complex process, involving multiple cell types and signaling mechanisms that inhibit or even propagate tumor growth. The interaction between tumor cells and the immune system relies on a conceptual framework of tumor immunoediting, a sequence of tumor suppression, homeostasis, and subversion/evasion.^2^ A vital, but not fully understood feature in this process is the presence of tumor infiltrating immune cells.^3^ In breast cancer, immune cell infiltrates can constitute as much as half of the tumor mass in patients, and be comprised of many different types of immune cells.^4^ The focus of studies in breast patients has been limited to the prognostic effect of tumor invasive lymphocytes (TIL) cell types,^5^ although there is also increasing evidence to support the relevance of other immune cell types in breast cancer.^6^, ^7^

Breast cancer is a heterogeneous disease, made up of 4 distinct receptor subtypes Luminal A, Luminal B, human epidermal growth factor receptor 2 positive (Her2+), and triple negative (TNBC). Clinical trials evaluating adjuvant therapy and immune invasiveness found that the presence of TILs in TNBC and Her2+ receptor subtypes is associated with improved treatment response and survival.^5^ In previous research, both Her2+ and TNBC tumors had higher TIL and were more immune‐rich than hormone receptor‐positive (Luminal A and Luminal B) tumors.^8^ However, the picture of how tumor‐invasive immune cells and receptor subtypes are related in breast cancer remains incomplete. Reviews summarizing the association of TIL infiltration with survival in breast tumors show that outcomes depend on how immune cells are measured, which cell types are included, and how they modify the pathogenesis in different subtypes.^9^, ^10^

There is no gold standard for the measure of lymphocytes and other tumor invasive immune cell types. In clinical settings, the recommended methodology for classifying tumors into immune‐poor/rich remains hematoxylin and eosin (H&E) staining of tumor sections.^11^ Noninfiltrative, immune‐poor tumors have been identified as a stronger predictor of survival than any invasive cell type in estrogen receptor negative (ER‐) tumors.^12^ Thus, the ability to reliably identify and better understand the functional states of immune‐poor tumors is of high translational relevance for a large subset of patients, yet it remains a missing gap in the current knowledge of breast tumor immune microenvironment.

The goal of this research study is to use three independent sets of data to create a validated, standard signature of tumor invasive immune cells genes. We aim to better understand how the presence/lack of immune cell infiltration in a primary tumor relates to distinct molecular/pathologic features of disease, specifically receptor subtype, and lymph node metastasis. Rather than focus on the relative abundance of each invasive immune cell type in tumors, we use a standard gene expression array‐based signature of 547 genes to create a binary (immune‐rich or immune‐poor) variable of immune invasion status across three large breast cancer cohorts. Among patients with immune‐rich tumors, we further identify the specific signature genes on each subtype. Lastly, we apply pathway analysis to identify important molecular mechanisms behind both immune‐rich and immune‐poor tumor groups.

## 2 MATERIALS AND METHODS

### 2.1 Study patients

We evaluated patient data from three independent publicly available data sources, final data for analysis includes: 650 patients from The Cancer Genome Atlas (TCGA),^13^ 772 patients from the Molecular Taxonomy of Breast Cancer International Consortium (METABRIC) ^14^ and 947 patients from Gene Expression Omnibus (GEO) compiled datasets ^15^ (Table 1). Lymph node metastasis at diagnosis was used as a binary endpoint (yes/no), and receptor subtype (based on hormone receptor status) was included as the only variable common to all sets of data. In order to maximize inclusion of patients with missing progesterone (PR) or estrogen receptor (ER) data, breast subtypes of Luminal A and Luminal B were combined into a single, Luminal variable, thus, the receptor subtype used across datasets are: Luminal, triple negative receptors (TNBC) and Her2+ (HER). Tumor histologic type was an inclusion criterion and breast tissue not classified as invasive ductal carcinoma (IDC) was excluded; (in TCGA 236 samples were excluded and 429 in METABRIC). In addition, any patient with missing stage information was excluded to avoid misclassification of ductal carcinoma in‐situ (DCIS), noninvasive tumors (METABRIC only *n* = 774). GEO data had no information regarding histologic tissue type, yet all patients were confirmed non‐DCIS using size and grading information.

**TABLE 1:** Association between tumor immune cell signature status NM and receptor subtype for across datasets

Immune‐signature	METABRIC			GEO			TCGA		
	Poor (*N*=639)	Rich (*N*=133)	P	Poor (*N*=482)	Rich (*N*=465)	P	Poor (*N*=327)	Rich (*N*=323)	P
Nodal Metastasis			*ns*			*ns*			*ns*
negative	328 (51.3%)	61 (45.9%)		323 (68.7%)	297 (65.0%)		136 (41.6%)	157 (48.6%)	
positive	311 (48.7%)	72 (54.1%)		147 (31.3%)	160 (35.0%)		191 (58.4%)	166 (51.4%)	
Receptor subtype			*			*			*
missing	2 (0.3%)	0 (0.0%)		–	–		17 (5.2%)	35 (10.8%)	
HER2	83 (13.0%)	19 (14.3%)		47 (12.6%)	74 (19.6%)		10 (3.1%)	20 (6.2%)	
Luminal	445 (69.6%)	60 (45.1%)		272 (72.9%)	189 (50.0%)		278 (85.0%)	210 (65.0%)	
TNBC	109 (17.1%)	54 (40.6%)		54 (14.5%)	115 (30.4%)		22 (6.7%)	58(18.0%)	

Note

P: *p*‐value ‐ *Indicates *p* < 0.001, *ns* indicates not significant at the 0.05 significance threshold

### 2.2 RNA expression data

METABRIC used the Illumina HT‐12v3 platform in gene expression analysis.^14^ In TCGA, mRNA expression counts were derived from the TCGA Level 3 RNAseqV2 expression data collected using Illumina HiSeq 2000 platform.^13^ The GEO study data are a curated aggregate of six Affymetrix Human Genome HG‐U133A array datasets.^15^ Gene Expression Omnibus (http://www.ncbi.nlm.nih.gov/geo) and Array Express (http://www.ebi.ac.uk/arrayexpress) accession numbers are: E‐TABM‐158,^16^ GSE6532,^17^ GSE3494,^18^ GSE1456,^19^ GSE7390,^20^ and GSE2603.^21^ All RNA intensities and read counts were either available at, or converted to gene level measurements. Based on the leukocyte specific gene signature matrix (LM22) immune cell type data, 515 of 547 immune cell specific genes were present in all three sets of patient data. The expression values for each dataset were independently standardized and combined results were evaluated for consistency using standard cutpoints and filtering conditions.

### 2.3 Primary tumor immune–rich/immune‐poor cell activity

We estimated immune cells abundance in the tumor sample, derived from the LM22 signature employing the immune cell expression profiling using CIBERSORT algorithm.^22^ This LM22 signature is comprised of 547 genes, which represent unique expression values for 22 specific human leukocyte cell types. LM22 has been validated in a process involving variably pure leukocyte subsets as well as bulk tumors from multiple cancer types, including breast cancer.^22^ This approach has been shown to be effective in identifying non‐tumor cells through comparison to flow sorted validation measures in follicular lymphoma.^23^ In breast cancer, leukocyte cell type specific expression has been confirmed as an accurate measure of tumor immune infiltrates.^24^

CIBERSORT, based on the LM22 gene signature, was used to estimate immune cells infiltrating the tumor.^22^ CIBERSORT’s core algorithm employs a machine learning approach to derive infiltrating cell types from a patient's whole‐transcriptome RNA tumor expression. Rather than quantifying the proportion of tumor infiltrating cell types, we applied the first part of CIBERSORT’s algorithm to categorize the overall immune‐rich/immune‐poor status of a tumor. Briefly, for each tumor sample, genes (the same number as LM22 signature genes) are randomly chosen from the full transcriptome. The correlation between random tumor RNA and signature RNA values was tested using a Pearson product‐moment correlation. This process was repeated 1000 times per sample to establish a distribution of null correlation values. The Pearson's correlation coefficient for the real test of tumor RNA to signature RNA was then ranked among the 1000 random test statistics for a *p*‐value. Samples under the *p* = 0.05 threshold were termed immune‐rich.

### 2.4 Association testing and gene set tests

Both METABRIC and TCGA had 0.3% and 8% missing subtype information, respectively. Differential expression analysis, for immune‐rich/poor phenotype, was conducted with linear models using the R package limma.^25^ Empirical Bayes ^26^ shrinkage was used to conduct t‐statistics for each gene and multiple comparisons were adjusted with the Benjamini‐Hochberg correction.^27^ In TCGA, raw counts of RNA per‐gene were compiled across the transcriptome of each patient and then assembled into a gene‐by‐patient matrix. Normalization factors for the raw data matrix were calculated, and a negative binomial model of differential counts was used in the edgeR package.^28^ Over‐representation analysis (OVA) for up or downregulated genes from DE models was performed using KEGG (Kyoto Encyclopedia of Genes and Genomes, http://www.genome.jp/kegg/) pathways and the limma “kegga” function.^25^ The scope of OVA analysis is intended to validate the variable of immune‐rich through any association with immune‐related pathways, and as a discovery approach for immune‐poor pathways.

#### 2.4.1 Differential expression analysis

We first tested if there were genes with differential expression in the following categories: immune‐rich and immune‐poor and nodal metastasis status and breast cancer receptor subtype. We then evaluated if expression in LM22 signature genes was changed in immune‐rich samples across the conditions of subtype or nodal status. Differential expression (DE) among immune‐rich and ‐poor tumors was assessed within each subtype and compared against all other subtypes (grouped together, not pairwise). Results lists from each source of patient data (METABRIC, GEO, and TCGA) were compared against one another for validation, and only intersecting genes with association FDR‐adjusted *p* < 0.05 and log fold change in the same direction were considered. Since LM22 is used to identify primarily immune‐rich tumors, we conducted a secondary, genome‐wide, DE analysis excluding LM22 (non‐LM22) genes in order to identify any shared genes expressed in immune‐poor groups, and to check for additionally over expressed non‐LM22 genes in immune‐rich tumors. All LM22 genes were excluded in this testing, and results were filtered/validated according to FDR adjusted *p* < 0.05 and log fold change in the same direction.

### 2.5 RESULTS

### 2.6 Tumor immune signature groups

Receptor subtype was associated with both nodal status and immune‐rich/poor tumors. Approximately 61% (1454/2369) of tumors in all datasets were Luminal type. Luminal patient tumors were more frequently immune‐poor in all data. Conversely, TNBC and Her2+ patients consistently had more immune‐rich tumors across all datasets (Table 1). No association was found between nodal metastasis status and immune‐poor/rich tumor groups in any of the three datasets (Table 1 **)**. In addition, no definitive connection between NM and immune‐cell signature was identified in LM22 signature gene analysis (data not shown).

### 2.7 mRNA differential association between immune phenotypes

While overall immune‐rich/poor status differed significantly across receptor subtype, individual signature genes seemed to be the same across the three subtypes, suggesting that the same genes responsible for immune infiltration do not differ across subtype. There were 10 total LM22 signature genes with consistently higher expression in immune‐rich tumors (FDR *p* < 0.05, see Table 2). However, the Her2 subtypes sample sizes were too small for estimates (*n* = 30 in TCGA), several of the TNBC only genes were not significant, most likely due to lack of power. TNBC receptor subtype showed no unique differences in genes associated with immune‐rich status when compared to all receptor subtypes. Presence of lymph node metastasis had no consistent effect upon tumor immune‐rich/poor status across datasets (analysis not shown).

**TABLE 2:** Differentially expressed LM22 genes between immune‐rich and immune‐poor tumors, for all patients, and receptor‐stratified

Gene	Log Fold Change (Log FC)								
	All receptor subtypes			Luminal type only			TNBC type only		
	(M)	(G)	(T)	(M)	(G)	(T)	(M)	(G)	(T)
*CCL19*	2.09	1.77	1.24	2.08	1.54	1.01	2.02	1.36	1.60
*CCL5*	1.77	1.35	1.18	1.73	1.32	1.01	1.31	1.15	1.05
*CD2*	1.76	1.01	1.05	*ns*	*ns*	*ns*	*ns*	*ns*	*ns*
*CD3D*	1.88	1.00	1.22	1.80	1.00	1.10	*ns*	*ns*	*ns*
*CXCL10*	1.68	1.39	1.55	1.67	1.12	1.01	*ns*	*ns*	*ns*
*CXCL9*	2.29	3.10	1.83	2.31	2.70	1.76	1.50	2.69	1.24
*GZMA*	1.55	1.14	1.12	1.46	1.14	1.01	*ns*	*ns*	*ns*
*GZMK*	1.93	1.17	1.12	1.92	1.25	1.07	*ns*	*ns*	*ns*
*MMP9*	1.26	1.28	1.50	1.16	1.45	1.58	*ns*	*ns*	*ns*
*SELL*	1.34	1.20	1.48	*ns*	*ns*	*ns*	*ns*	*ns*	*ns*

#### 2.7.1 LM22 immune‐rich differences across receptor subtypes

Within immune‐rich tumors only, differential expression of genes was observed between both Luminal and TNBC subtypes. Nine LM22 genes had significant differential expression (FDR adjusted *p* < 0.05) and consistent direction of log fold change across the three datasets, (7 in TNBC and 2 in Luminal with respect to the other subtypes). Both *KYNU* and *EPHA1* transcripts were significantly decreased in the Luminal immune‐rich tumor group. In TNBC patients with immune‐rich samples, expression of *CHI3L2* and *FES* was significantly upregulated relative to all other subtypes. Decreases of expression in *TRPM*, *ABCB9*, and *EPHA1* were unique to the TNBC immune‐rich set of patients (Table 3). No consistent differences were found in the Her2 receptor subtype.

**TABLE 3:** Top LM22 genes differentially expressed by subtype within immune‐rich tumors

Gene	METABRIC		GEO		TCGA		
	Log FC (M)	FDR *p*‐value (M)	Log FC (G)	FDR *p*‐value (G)	Log FC (T)	FDR *p*‐value (T)	
**Subtype**							
**Luminal vs other**						
*KYNU*	−1.02	<0.001	−3.12	<0.001	−1.82	<0.001	
*EPHA1*	−0.64	<0.001	−0.11	0.01	−0.76	<0.001	
**TNBC vs other**							
*CHI3L2*	1.18	<0.001	0.84	<0.001	2.10	<0.001	
*FES*	0.74	<0.001	0.09	0.01	0.77	0.01	
*FFAR2*	−0.35	0.03	−0.39	<0.001	−2.09	<0.001	
*TRPM4*	−0.53	<0.001	−0.23	<0.001	−1.42	<0.001	
*FAM174B*	−0.97	<0.001	−0.22	0.01	−1.27	<0.001	
*ABCB9*	−0.60	0.03	−0.16	<0.001	−1.07	<0.001	
*EPHA1*	−0.60	<0.001	−0.10	0.04	−0.61	0.05	

Abbreviation: Log FC =Log Fold Change (subtype/all other subtypes).

#### 2.7.2 Non‐LM22 DE and pathway analysis

**FIGURE 1:**
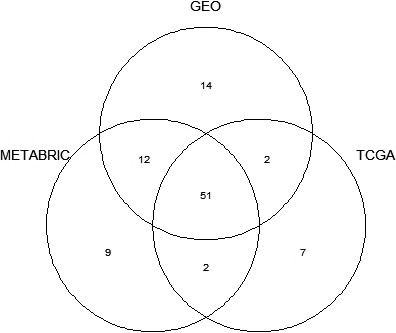
Upregulated pathways in over‐representation analysis (OVA) and overlap among datasets

**FIGURE 2:**
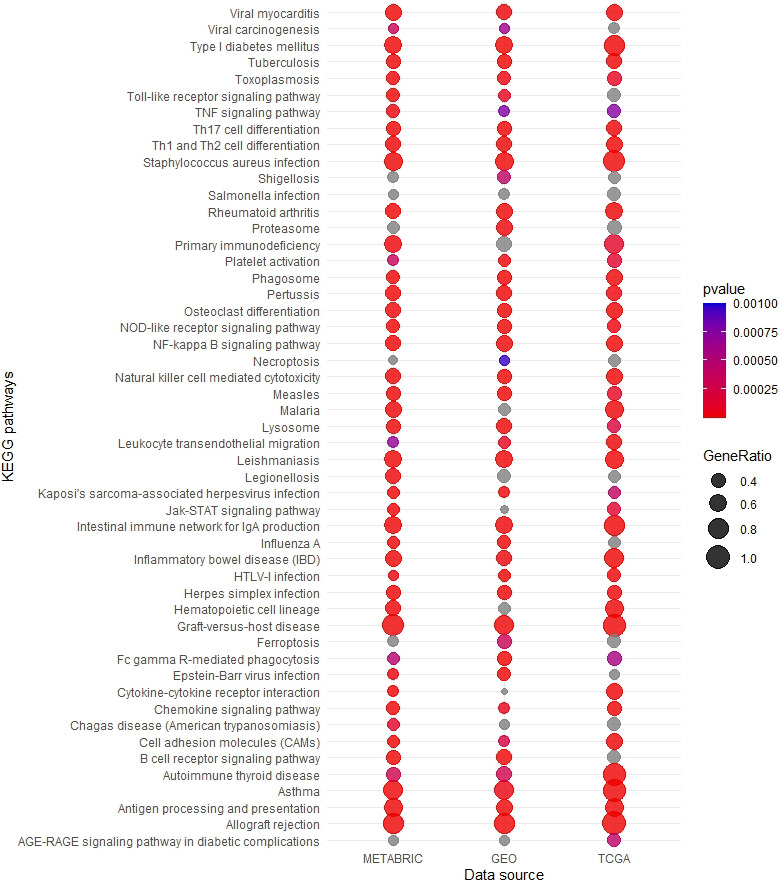
Over‐representation analysis results of 51 upregulated KEGG pathways in immune‐rich tumor expression. Gene ratio is number of upregulated genes/total genes in pathway

Excluding all LM22 signature genes, we performed a whole transcriptome analysis of differential expression between immune‐rich and immune‐poor tumors, adjusting for receptor subtype and nodal metastasis. Differential expression analysis identified 1,951 total genes shared across datasets with expression associated to immune‐rich status. Of these results, nine genes showed statistically significant DE (FDR adjusted *p* < 0.05) between the 2 immune phenotypes consistently across the three datasets (Table 4). The expression of *GRIA2* and *SCUBE2* genes was associated with immune‐poor tumors. Importantly, gene OVA applied to the models above found no KEGG pathways to be consistently upregulated in immune‐poor tumors. In tumors with an immune‐rich signature, 51 sets of KEGG pathways had significantly over‐represented, and upregulated genes in all datasets (Figure 1). Over‐represented pathways with the strong, consistent associations across datasets included; antigen processing and presentation, natural killer cell mediated cytotoxicity, NOD‐like receptor signaling pathway, and various immune response to infection (Figure 2).

**TABLE 4:** Expression of top non‐LM22 signature genes: immune‐rich versus immune‐poor tumors

Gene	Log FC (METABRIC)	Log FC (GEO)	Log FC (TCGA)
*C1QB*	1.16**	0.81**	1.34**
*CD52*	1.89**	1.06**	2.63**
*CORO1A*	1.15**	0.82**	1.37**
*EVI2B*	1.42**	0.81**	1.36**
*GBP1*	1.02**	0.97**	1.47**
*LYZ*	1.83**	1.01**	2.05**
*PSMB9*	1.17*	0.84**	1.21**
*GRIA2*	−0.58**	−2.35*	−1.30**
*SCUBE2*	−0.57**	−0.54*	−1.21**

Abbreviation: Log FC =Log Fold Change, **FDR *p* < 0.0001, *FDR *p* < 0.01.

## 3 DISCUSSION

Our approach classified 2369 patients into two groups (immune‐rich, and immune‐poor) using an immune cell expression signature. The overall frequency of immune‐rich versus immune‐poor tumors differed by receptor subtype; however, the specific signature genes remained the same, suggesting that signature expression is a stronger determinant of immune infiltration than receptor subtype. Common to both TNBC and Luminal patients, the genes for C‐C motif chemokine ligand genes of *CCL5* and *CCL19* as well for the C‐X‐C motif chemokine *CXCL9* exhibited the highest differential expression in immune‐rich tumors compared to immune‐poor tumors, regardless of subtype. Granzyme genes encoding *GZMA* and *GZMK* were consistently high across cohorts of Luminal patients but not for TNBC. While Luminal tumors had the smallest proportion of immune‐rich tumors, they also had the more significantly different LM22 signature genes than TNBC. This non‐intuitive finding is partially due to the large overall proportion of breast tumors that are Luminal, adding more sample size and power for DE analysis. In immune‐rich only tumor analysis, no significant increases in the expression of LM22 genes was found in Luminal versus all other subtypes. TNBC patients had the highest percentage of immune‐rich versus immune‐poor tumors per subtype. Both *CHI3L2* and *FES* showed TNBC‐differential over expression in immune‐rich subset analysis. Recent breast cancer models in mice have also indicated increased *FES* expression as linked to tumor aggressiveness, macrophage infiltration, and involvement in the JAK3‐Fes‐PLD2 signaling pathway.^29^, ^30^, ^31^, ^32^ Chitinase 3‐like‐2 (*CHI3L2*) has been identified as a marker of M2 tumor associated macrophages, yet its effect on tumor progression in breast cancer is not fully known.^33^, ^34^

We also observed several new relationships between non‐LM22 genes and immune‐rich tumors, specifically overexpression of *LYZ*, *C1QB*, *CORO1A*, *EVI2B*, *GBP1*, *PSMB9*, and *CD52* in immune‐rich tumors independent of receptor subtype or nodal metastasis. Likewise, overexpression of both *SCUBE2* and *GRIA2* was consistently associated with immune‐poor status across datasets. Interestingly, *SCUBE2* has been associated with ER/PR positive tumors ^35^ and proposed as a tumor suppressor in breast cancer,^36^ yet it has also been shown as the only common gene in outcome‐predictive signature assays in breast cancer.^37^ The gene *GRIA2* is a member of a family of glutamate receptors, and its expression has been linked to the modulation of both tumor and immune cell function.^38^ We identified several novel markers which were overexpressed in immune‐rich tumors. CAMPATH‐1 antigen (*CD52*) is expressed in mature lymphocytes. It is the target of the monoclonal antibody therapy alemtuzumab in several diseases including chronic lymphocytic leukemia, cutaneous T‐cell lymphoma (CTCL), and multiple sclerosis (MS).^39^ In breast cancer stromal tissue, *CD52* has been linked to an expression signature of poor outcome,^40^, ^41^ our results give further evidence of the potential of its overexpression as marker of immune cell invasion in breast tumors.

One of the genes most differentially expressed between immune‐rich and immune‐poor tumors was lysozyme (*LYZ*). Whether increased lysozyme production originates from normal or cancerous breast cells or from invasive immune cells, or both, is less clear. Lysozyme is secreted by neutrophils and macrophages in inflammatory response,^42^ and is also present in the breast secretions of ~98% of healthy non‐lactating women.^43^ Previous research of the breast milk protein in breast tumors showed an inverse relationship between cancer severity and increased expression of *LYZ*.^44^ In the immune response, lysozyme degrades the bacterial cell wall with components that activate molecules downstream of pattern recognition receptors such as inflammasomes, Toll‐like receptors, and NOD‐like receptors.^45^ In OVA, NOD‐like receptor signaling was upregulated in invasive immune cells, particularly the guanylate binding proteins of *GBP1*, *GBP4*, and *GBP5*. GBP‐1 has been associated with various antimicrobial activities as well as paclitaxel resistance in cell lines of some tumors with the notable exception of breast.^46^ While its contributions to prognostic factors across cancer types is varied, *GBP1* has been associated with improved survival in breast cancer.^47^

There was no clear association between our signature of tumor immune cell infiltration and the extrinsic phenotype of nodal metastasis status. No single gene result from differential expression testing was both significant and consistent in direction of association across datasets. In addition, no interaction by subtype was observed which might have obscured the association between NM and RNA expression by immune signature group. These results could suggest that while draining lymph nodes are a vital part of the antitumor immune response, the process of lymph node metastasis in breast cancer is not demonstrably linked to the infiltration of tumor invasive immune cells. Importantly, our results of LM22 genes being associated with immune‐poor phenotype are expected, given the use of LM22 in classifying immune cells. Furthermore, we found no consistently over/under represented pathways for immune‐poor tumors, and only 2 non‐LM22 genes were significantly upregulated in immune‐poor analysis.

In summary, our analysis identified several signature tumor transcripts associated with immune‐rich and immune‐poor status. Future research on the topic may be informed by the finding that, while there are individual immune‐rich genes which are uniquely expressed in TNBC and Luminal tumors, the majority of validated, immune‐rich gene expression is not affected by receptor subtype or nodal metastasis. Additionally, these data provide two potential novel targets for the study of the immune microenvironment of immune‐poor tumors; *SCUBE2* and *GRIA2*.

## Conflict of Interest

None.

## AUTHORS’ CONTRIBUTIONS

**Conception and design:** M. Behring, S. Shrestha, A.I. Vazquez, **Development of methodology:** M Behring, A.I. Vazquez, **Analysis and interpretation of data (e.g., statistical analysis, biostatistics, computational analysis):** M. Behring, S. Shrestha, A.I. Vazquez, **Writing, review, and/or revision of the manuscript:** M. Behring, Y. Ye, A. Elkholy, P. Bajpai, S. Agarwal, H. Kim, H. W. Wiener, A. I. Ojesina, S. Shrestha, U. Manne, A.I. Vazquez, **Study supervision:** S. Shrestha, U. Manne, A.I. Vazquez.
